# Construction of prognostic models for colorectal cancer based on the perioperative modified Gustave Roussy Immune Score and machine learning

**DOI:** 10.3389/fimmu.2026.1761650

**Published:** 2026-07-30

**Authors:** Jie Zhou, Kexin Zhao, Shaozhong Wei, ShuaiJin Huang

**Affiliations:** 1 Department of Medical Oncology, Cancer Center, West China Hospital, Sichuan University, Chengdu, China; 2 Department of Gastrointestinal Oncology Surgery, Hubei Cancer Hospital, Tongji Medical College, Huazhong University of Science and Technology, Wuhan, Hubei, China; 3 Department of Hematology, The Second Xiangya Hospital, Central South University, Changsha, Hunan, China; 4 Department of Emergency, Hunan Provincial Hospital of Integrated Traditional Chinese and Western Medicine, Changsha, Hunan, China

**Keywords:** colorectal cancer, dynamic mGRIm trajectory, machine learning, modified Gustave Roussy Immune Score, prognosis

## Abstract

**Objective:**

To evaluate the prognostic value of preoperative and postoperative modified Gustave Roussy Immune Score (mGRIm) and dynamic mGRIm trajectories in patients with colorectal cancer (CRC), and to develop internally validated machine-learning models based on perioperative dynamic features.

**Methods:**

This retrospective cohort study included 457 patients with CRC who underwent primary tumor resection at Hubei Cancer Hospital between January 2016 and December 2019. Preoperative and postoperative mGRIm scores were calculated using receiver operating characteristic curve-derived cut-off values for albumin, lactate dehydrogenase, and neutrophil-to-lymphocyte ratio. Dynamic mGRIm trajectory was defined as the transition between preoperative and postoperative mGRIm risk states. Associations with overall survival (OS) and disease-free survival (DFS) were evaluated using Kaplan–Meier analysis and Cox proportional hazards models. Machine-learning model development incorporated Boruta-based feature selection, training set–restricted 5-fold cross-validation, and training set–restricted resampling strategies.

**Results:**

Dynamic mGRIm trajectory showed strong and consistent prognostic stratification. Patients with persistently high mGRIm status had significantly higher risks of death (HR = 3.47, 95% CI: 1.76 -6.83, p < 0.001) and DFS events (HR = 2.76, 95% CI: 1.65 -4.61, p < 0.001) than those with persistently low status. In the prespecified evaluation framework integrating multiple performance metrics, the neural network model achieved the highest composite score for OS prediction (AUC = 0.801), whereas the radial basis function support vector machine achieved the best performance for DFS prediction (AUC = 0.856) in the independent test set.

**Conclusion:**

Dynamic mGRIm trajectory provides robust prognostic information for patients with CRC. Machine-learning models based on perioperative dynamic features demonstrate satisfactory internal predictive performance; however, external validation is required prior to clinical application.

## 1 Introduction

Colorectal cancer is a highly prevalent malignancy of the digestive system, and its global disease burden continues to increase. Epidemiological data show that colorectal cancer ranks third in global cancer incidence (approximately 10.0%) and second in cancer-related mortality (9.4%) (1). In 2020 alone, more than 1.9 million new cases and 935,000 deaths were reported worldwide (1). The pathogenesis of colorectal cancer is multidimensional and involves complex interactions among genetic, sociodemographic, medical, environmental, lifestyle, and dietary factors (2). Previous studies have identified behavioral factors such as smoking, alcohol abuse, obesity, sedentary lifestyle, and excessive red meat intake as important contributors (3). Current clinical treatment is centered on radical surgery, with chemotherapy and radiotherapy used as adjunctive treatments when appropriate. However, approximately half of patients respond poorly to conventional treatment, resulting in high rates of postoperative recurrence and metastasis (4). Although targeted therapy and immune checkpoint inhibitors have prolonged survival in selected subgroups, the development of individualized treatment strategies remains challenging (5–7).

For prognostic assessment, the TNM staging system remains the cornerstone of risk stratification in colorectal cancer; however, substantial heterogeneity in survival outcomes is still observed among patients within the same stage (8). This heterogeneity highlights the need to integrate additional biomarkers into more accurate prediction models. In recent years, increasing attention has been paid to systemic inflammation and nutritional status, and combinations of peripheral blood indicators, such as the neutrophil-to-lymphocyte ratio and platelet-to-lymphocyte ratio, have shown prognostic value (9, 10). In particular, the Gustave Roussy Immune Score (GRIm score), which integrates lactate dehydrogenase (LDH), serum albumin (ALB), and the neutrophil-to-lymphocyte ratio (NLR), has demonstrated prognostic value across several solid tumors (11–15).

The clinical use of the GRIm score has continued to evolve. Improved versions have been developed for specific tumor contexts. For example, the HCC-GRIm score for hepatocellular carcinoma incorporates liver-specific indicators, including the AST/ALT ratio and total bilirubin, to improve prognostic performance (12). Other studies have used receiver operating characteristic curves to determine data-driven cut-off values for individual parameters and construct a more adaptable mGRIm scoring system (14, 16). However, most existing studies have assessed GRIm or mGRIm at a single time point, and the prognostic value of perioperative mGRIm risk-status trajectories remains insufficiently explored. Although dynamic changes in the GRIm score during nivolumab treatment have been reported to predict treatment efficacy (17), evidence in the surgical oncology setting remains limited.

This study focused on the dynamic evolution of mGRIm during the perioperative period in patients with resectable CRC. We aimed to systematically evaluate the prognostic value of preoperative mGRIm, postoperative mGRIm, and dynamic mGRIm trajectory. By integrating perioperative dynamic features into machine-learning models, this study sought to provide evidence that may help refine prognostic risk stratification for surgical patients. The findings may also support further investigation of perioperative biomarkers in CRC, while requiring external validation before clinical implementation.

## 2 Materials and methods

### 2.1 Study population

This retrospective cohort study included patients with histopathologically confirmed primary colorectal cancer who underwent radical surgery for the primary tumor at Hubei Cancer Hospital, affiliated with Huazhong University of Science and Technology, between January 2016 and December 2019. The exclusion criteria were as follows: (i) history of other primary malignancies; (ii) age <18 years or unclear age at diagnosis; (iii) follow-up <1 month or missing key follow-up data; (iv) systemic diseases or related medication use that could affect blood test indicators; and (v) incomplete perioperative records or clinicopathological data. A total of 719 patients who underwent radical surgery for primary colorectal cancer at Hubei Cancer Hospital between January 2016 and December 2019 were initially screened. After excluding patients with a history of other primary malignancies (n = 3), age <18 years or unclear age at diagnosis (n = 5), follow-up <1 month or missing key follow-up data (n = 40), systemic diseases or related medication use that could affect blood test indicators (n = 17), and incomplete perioperative records or clinicopathological data (n = 197), 457 patients were finally included in the analysis. The detailed patient selection process is shown in **Supplementary Figure 1**. The study followed the ethical principles of the Declaration of Helsinki. The study protocol was approved by the Medical Ethics Committee of Hubei Cancer Hospital, and all clinical data were anonymized before analysis.

### 2.2 Data collection

Four categories of clinical data were systematically collected through retrospective review of the electronic medical record system of Hubei Cancer Hospital (LLHBCH2025YN-023): demographic characteristics, tumor biological characteristics, laboratory indicators measured within one week before surgery and before postoperative adjuvant therapy, and postoperative treatment information. All data were extracted in a structured manner and quality-controlled using a standardized process to ensure completeness and reliability.

### 2.3 Definition and grouping criteria for mGRIm score

NLR was calculated as the neutrophil-to-lymphocyte ratio. The optimal cut-off values for preoperative and postoperative ALB, LDH, and NLR were determined by receiver operating characteristic curve analysis using the Youden index, with overall survival status as the classification outcome. The corresponding cut-off values, AUCs, sensitivity, specificity, and Youden indices are shown in **Supplementary Table 4**. For score construction, ALB below the cut-off and LDH or NLR above the cut-off were each assigned 1 point.

As shown in **Supplementary Figure 2**, the preoperative and postoperative mGRIm scores were calculated separately by summing the three component scores, with total scores ranging from 0 to 3. Scores ≤1 were classified as low mGRIm, whereas scores >1 were classified as high mGRIm. To characterize perioperative changes, a dynamic mGRIm trajectory was defined according to the combination of preoperative and postoperative mGRIm categories: group I, both low; group II, either preoperative or postoperative high; and group III, both high.

### 2.4 Study endpoints and follow-up

The study used two endpoints: overall survival (OS) and disease-free survival (DFS). OS was defined as the time from surgery to all-cause death or last follow-up, whichever occurred first. DFS was defined as the time from surgery to the first occurrence of local recurrence, distant metastasis, all-cause death, or last follow-up. Survival data were collected using a multi-source follow-up strategy that combined electronic medical record review with structured telephone follow-up. The observation period extended from the date of surgery to the predefined study endpoint (September 1, 2022) or the occurrence of an endpoint event. Follow-up intervals were standardized according to National Comprehensive Cancer Network (NCCN) guidelines.

### 2.5 Research methodology

#### 2.5.1 Statistical methods

This retrospective study did not include prospective sample size estimation. Survival curves were generated using the Kaplan-Meier method, and the log-rank test was used to compare overall survival and disease-free survival across groups with different mGRIm scores. Multivariable analyses were performed using Cox proportional hazards regression to calculate hazard ratios (HRs) and 95% confidence intervals (CIs), with potential confounders controlled through hierarchical modeling. To examine the robustness of the three-level dynamic mGRIm trajectory, sensitivity analyses were performed using alternative definitions of perioperative mGRIm change, including a binary trajectory definition, an ordinal trajectory definition, signed mGRIm change, and absolute mGRIm change. All sensitivity analyses were conducted using the fully adjusted Cox regression model. All statistical tests were two-sided, and p<0.05 was considered statistically significant. Data processing and analyses were performed using R software (version 4.3.4).

#### 2.5.2 Subgroup analysis

Subgroups were defined according to age, gender, lymphovascular invasion status, perineural invasion, and tumor marker levels. The associations of mGRIm scores with OS and DFS within these subgroups were assessed using multivariable Cox proportional hazards regression. Interaction analyses were performed using likelihood ratio tests, and forest plots were used to visualize the results.

#### 2.5.3 Predictive modeling and validation

Candidate machine-learning models were constructed using the dynamic feature set. Preoperative and postoperative mGRIm scores were retained for Kaplan–Meier survival analysis, Cox regression, and subgroup analysis, whereas machine-learning model construction focused on perioperative dynamic predictors.

The dataset was randomly divided into a training set and a testing set at a ratio of 7:3 using stratified sampling according to outcome status. Feature selection and model training were performed exclusively within the training set to reduce information leakage. Candidate predictors included clinicopathological variables and perioperative dynamic laboratory changes. Boruta feature selection was first performed in the training set, and the selected predictors were then used for machine-learning model construction. Boruta feature selection was performed in the training set using 500 trees and a maximum of 100 iterations. To assess the stability of selected predictors, the Boruta procedure was repeated across 100 bootstrap resampling runs within the training set, and the selection frequency of predictors confirmed in the primary Boruta analysis was calculated.

Thirteen candidate machine-learning algorithms were successfully fitted and compared, including neural network, averaged neural network, elastic net regression, support vector machine with radial basis function kernel, support vector machine with linear kernel, logistic regression, linear discriminant analysis, quadratic discriminant analysis, naive Bayes classifier, multivariate adaptive regression splines, random forest, extreme gradient boosting, and C5.0 decision tree algorithm. Hyperparameter tuning was performed within 5-fold cross-validation in the training set. To address outcome class imbalance, up-sampling was applied within the resampling procedure. The locked testing set was used only for final model evaluation.

Model performance was assessed using AUC, accuracy, sensitivity, specificity, precision, F1 score, Kappa, and balanced accuracy. The final model for each endpoint was selected according to a prespecified composite score calculated as follows: 0.40 × AUC + 0.25 × balanced accuracy + 0.20 × F1 score + 0.15 × sensitivity. The Boruta-selected predictors, full model performance metrics, and final model selection criteria are provided in **Supplementary Tables 5**–**8**. An overview of the machine-learning workflow is provided in **Supplementary Figure 5**.

#### 2.5.4 Model robustness and stability assessment

To further evaluate the robustness of the modeling framework, multiple stability analyses were conducted. First, the feature selection process based on the Boruta algorithm was repeated across 100 bootstrap resampling iterations within the training set. The selection frequency of each predictor was calculated to quantify its stability. Predictors consistently selected in a high proportion of resamples were considered robust contributors to the final predictive models (18, 19).

Second, hyperparameter tuning and up-sampling were performed within 5-fold cross-validation in the training set (20). Boruta feature selection and model fitting were restricted to the training set, while the locked test set was reserved exclusively for final evaluation.

Third, only predictors with stable selection patterns across resampling iterations were retained in the final dynamic feature set used for machine-learning model construction. This approach minimized the risk of overfitting and reduced model selection bias introduced by a single feature selection run (21).

Collectively, these procedures supported the stability of feature selection and improved the reproducibility of the predictive modeling framework.

## 3 Results

### 3.1 Baseline information

A total of 457 patients were included in this study, and their clinicopathologic characteristics are shown in **Table 1**. Age (p=0.012), degree of differentiation (p=0.036), TNM stage (p<0.001), and positive circumferential resection margin (p=0.001) were significantly associated with OS. Degree of differentiation (p=0.004), TNM stage (p<0.001), type of pathology (p=0.002), lymphovascular invasion (p=0.013), perineural invasion (p=0.002), and adjuvant chemotherapy (p=0.035) were significantly associated with DFS. **Supplementary Tables 1**–**3** summarize the associations of the included preoperative and postoperative characteristics and perioperative dynamic variables with OS and DFS.

**Table 1:** Clinicopathologic characteristics stratified by survival and disease progression status.

Characteristic	Survival status		p-value	Progress status		p-value
	Alive (N = 369)	Dead (N = 88)		No (N = 309)	Yes (N = 148)	
Age, years	57.55 (± 10.60)	60.74 (± 10.53)	0.012	57.79 (± 10.39)	58.96 (± 11.16)	0.271
Gender			0.219			0.161
Male	236 (64.0%)	63 (71.6%)		195 (63.1%)	104 (70.3%)	
Female	133 (36.0%)	25 (28.4%)		114 (36.9%)	44 (29.7%)	
Smoke			0.996			0.903
Yes	119 (32.2%)	29 (33.0%)		99 (32.0%)	49 (33.1%)	
No	250 (67.8%)	59 (67.0%)		210 (68.0%)	99 (66.9%)	
Drink			0.995			0.407
Yes	87 (23.6%)	21 (23.9%)		69 (22.3%)	39 (26.4%)	
No	282 (76.4%)	67 (76.1%)		240 (77.7%)	109 (73.6%)	
BMI, kg/m²	22.92 (± 3.54)	22.38 (± 3.59)	0.204	22.88 (± 3.59)	22.67 (± 3.48)	0.549
Location			0.188			0.715
Left	73 (19.8%)	23 (26.1%)		62 (20.1%)	34 (23.0%)	
Right	62 (16.8%)	9 (10.2%)		50 (16.2%)	21 (14.2%)	
Rectum	234 (63.4%)	56 (63.6%)		197 (63.8%)	93 (62.8%)	
Differentiation			0.036			0.004
Low	48 (13.0%)	20 (22.7%)		36 (11.7%)	32 (21.6%)	
Medium	281 (76.2%)	63 (71.6%)		236 (76.4%)	108 (73.0%)	
High	40 (10.8%)	5 (5.7%)		37 (12.0%)	8 (5.4%)	
TNM			<0.001			<0.001
I	44 (11.9%)	1 (1.1%)		41 (13.3%)	4 (2.7%)	
II	148 (40.1%)	7 (8.0%)		130 (42.1%)	25 (16.9%)	
III	140 (37.9%)	35 (39.8%)		120 (38.8%)	55 (37.2%)	
IV	37 (10.0%)	45 (51.1%)		18 (5.8%)	64 (43.2%)	
Type of adenocarcinoma			0.266			0.002
non-mucinous	313 (84.8%)	74 (84.1%)		263 (85.1%)	124 (83.8%)	
mucinous	19 (5.1%)	8 (9.1%)		11 (3.6%)	16 (10.8%)	
mixed	37 (10.0%)	6 (6.8%)		35 (11.3%)	8 (5.4%)	
CRM			0.001			0.063
Positive	3 (0.8%)	6 (6.8%)		3 (1.0%)	6 (4.1%)	
Negative	366 (99.2%)	82 (93.2%)		306 (99.0%)	142 (95.9%)	
LVI			0.481			
Positive	109 (29.5%)	30 (34.1%)		82 (26.5%)	57 (38.5%)	0.013
Negative	260 (70.5%)	58 (65.9%)		227 (73.5%)	91 (61.5%)	
Perineural invasion			0.209			
Positive	83 (22.5%)	26 (29.5%)		60 (19.4%)	49 (33.1%)	0.002
Negative	286 (77.5%)	62 (70.5%)		249 (80.6%)	99 (66.9%)	
Chemotherapy			0.843			0.035
Yes	313 (84.8%)	76 (86.4%)		255 (82.5%)	134 (90.5%)	
No	56 (15.2%)	12 (13.6%)		54 (17.5%)	14 (9.5%)	
Radiotherapy			0.261			
Yes	16 (4.3%)	7 (8.0%)		12 (3.9%)	11 (7.4%)	0.163
No	353 (95.7%)	81 (92.0%)		297 (96.1%)	137 (92.6%)	

### 3.2 Kaplan-Meier survival analysis

**Figure 1:**
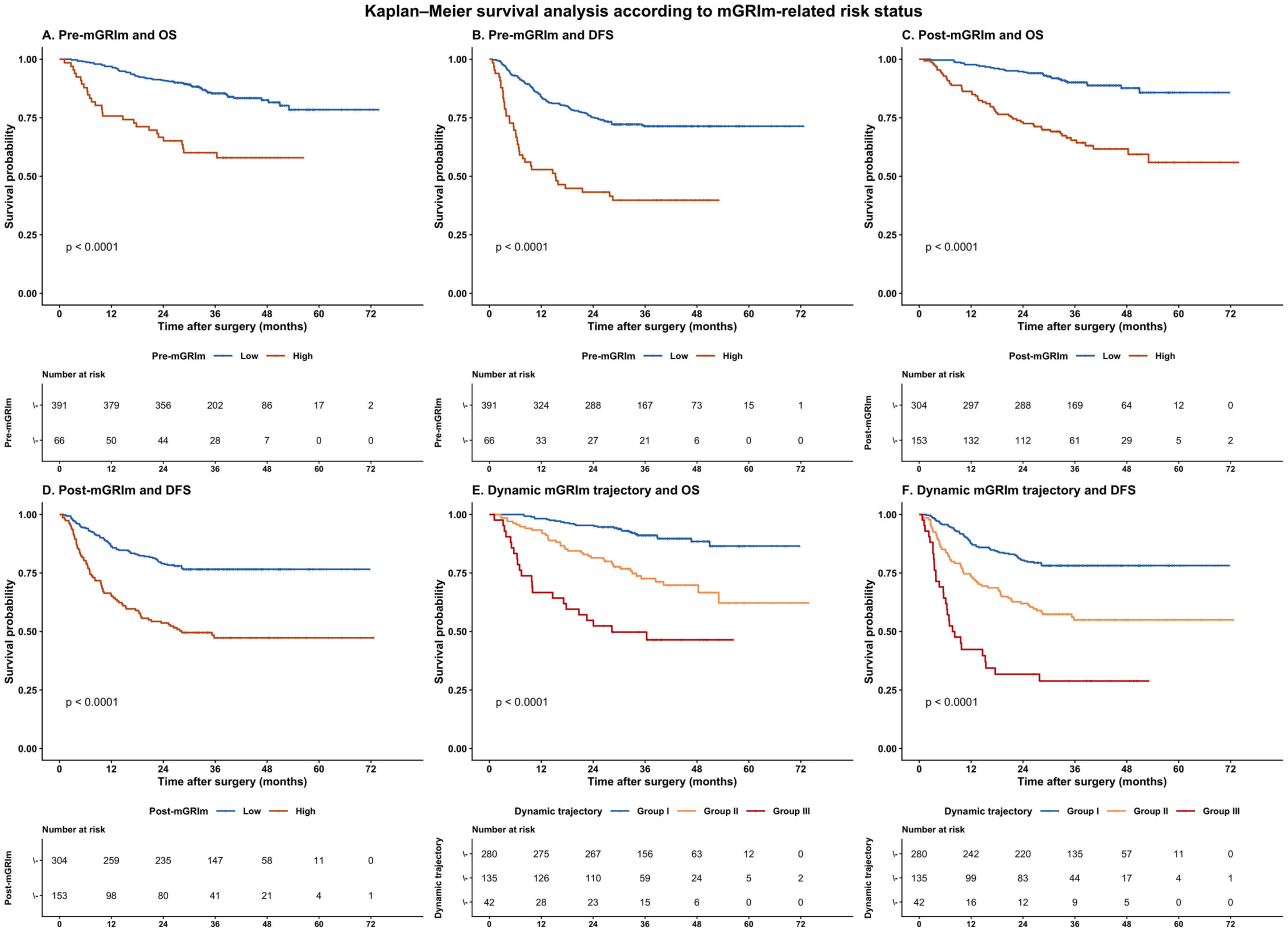
Kaplan–Meier survival analysis. **(A)** Preoperative mGRIm status and OS. **(B)** Preoperative mGRIm status and DFS. **(C)** Postoperative mGRIm status and OS. **(D)** Postoperative mGRIm status and DFS. **(E)** Dynamic mGRIm trajectory and OS. **(F)** Dynamic mGRIm trajectory and DFS. Group I, persistently low; Group II, either preoperative or postoperative high; Group III, persistently high. OS, overall survival; DFS, disease-free survival.

Kaplan-Meier survival analysis showed that preoperative mGRIm score, postoperative mGRIm score, and dynamic mGRIm trajectory were significantly associated with prognosis in patients with CRC (**Figure 1**). Specifically, higher mGRIm-related risk categories were associated with significantly poorer OS and DFS (p<0.001). The log-rank test further confirmed significant differences in survival curve distributions across mGRIm scoring groups, suggesting that the mGRIm scoring system has prognostic value.

### 3.3 Cox proportional hazards regression analysis

In the multivariable Cox proportional hazards regression models, preoperative mGRIm score, postoperative mGRIm score, and dynamic mGRIm trajectory were all significantly associated with OS and DFS after adjustment for potential confounders (**Table 2**). Among these variables, the dynamic mGRIm trajectory showed the most pronounced prognostic association.

**Table 2:** Multivariable Cox proportional hazards regression analysis of mGRIm-related variables for OS and DFS.

Variables	Model 1		Model 2		Model 3	
	HR (95% CI)	p-value	HR (95% CI)	p-value	HR (95% CI)	p-value
Pre-mGRIm score						
OS						
Low	1 [Reference]		1 [Reference]		1 [Reference]	
High	3.35 (2.10- 5.35)	<0.001	1.80 (1.09- 2.97)	0.021	1.96 (1.15- 3.34)	0.014
per 1 unit increase	2.10 (1.65- 2.66)	<0.001	1.60 (1.24- 2.05)	<0.001	1.62 (1.25- 2.11)	<0.001
DFS						
Low	1 [Reference]		1 [Reference]		1 [Reference]	
High	3.23 (2.21- 4.73)	<0.001	1.72 (1.14- 2.60)	0.009	1.86 (1.22- 2.84)	0.004
per 1 unit increase	1.94 (1.60- 2.34)	<0.001	1.47 (1.21- 1.79)	<0.001	1.49 (1.22- 1.82)	<0.001
Post-mGRIm score						
OS						
Low	1 [Reference]		1 [Reference]		1 [Reference]	
High	3.89 (2.49- 6.07)	<0.001	2.66 (1.68- 4.23)	<0.001	2.30 (1.42- 3.72)	<0.001
per 1 unit increase	2.13 (1.69- 2.68)	<0.001	1.74 (1.38- 2.19)	<0.001	1.62 (1.27- 2.08)	<0.001
DFS						
Low	1 [Reference]		1 [Reference]		1 [Reference]	
High	2.83 (2.03- 3.94)	<0.001	2.12 (1.49- 3.00)	<0.001	2.09 (1.46- 2.99)	<0.001
per 1 unit increase	1.95 (1.63- 2.34)	<0.001	1.68 (1.40- 2.02)	<0.001	1.66 (1.38- 2.01)	<0.001
Dynamic mGRIm trajectory						
OS						
Both times low	1 [Reference]		1 [Reference]		1 [Reference]	
Any one time high	3.11 (1.88- 5.13)	<0.001	2.47 (1.48- 4.11)	<0.001	2.11 (1.25- 3.55)	0.005
Both times high	8.14 (4.54- 14.61)	<0.001	3.37 (1.81- 6.26)	<0.001	3.47 (1.76- 6.83)	<0.001
DFS						
Both times low	1 [Reference]		1 [Reference]		1 [Reference]	
Any one time high	2.46 (1.70- 3.56)	<0.001	2.09 (1.43- 3.06)	<0.001	2.03 (1.38- 2.98)	<0.001
Both times high	6.04 (3.80- 9.59)	<0.001	2.56 (1.56- 4.18)	<0.001	2.76 (1.65- 4.61)	<0.001

Model 1: adjusted for gender, age, smoking, alcohol consumption, and BMI.

Model 2: adjusted for Model 1 + degree of differentiation + primary site + pathological type + TNM stage + lymphovascular invasion + perineural invasion.

Model 3: adjusted for Model 2 + adjuvant chemotherapy + postoperative radiotherapy + circumferential resection margin + lymph node metastasis rate.

In the fully adjusted model, compared with patients with persistently low mGRIm status, those with either preoperative or postoperative high mGRIm status had an increased risk of death (HR = 2.11, 95% CI: 1.25–3.55, p = 0.005), whereas patients with persistently high mGRIm status showed a further increased risk of death (HR = 3.47, 95% CI: 1.76–6.83, p < 0.001). Similar associations were observed for DFS. Compared with the persistently low group, the HR for DFS was 2.03 (95% CI: 1.38–2.98, p < 0.001) in patients with either preoperative or postoperative high mGRIm status and 2.76 (95% CI: 1.65–4.61, p < 0.001) in those with persistently high mGRIm status.

Sensitivity analyses further supported the prognostic relevance of the dynamic mGRIm trajectory. Alternative binary and ordinal definitions of perioperative mGRIm change were consistently associated with both OS and DFS, whereas simple signed or absolute arithmetic changes in mGRIm score were not significantly associated with prognosis (**Supplementary Table 9**). These findings suggest that the perioperative risk-status trajectory of mGRIm captures prognostic information beyond a simple numerical change in score. Therefore, subsequent machine-learning model construction focused on dynamic features.

### 3.4 Subgroup analysis

As shown in **Supplementary Figure 3**, the associations of preoperative and postoperative mGRIm status with OS and DFS were generally consistent across predefined subgroups. Similarly, when dynamic mGRIm trajectory was analyzed as any high versus persistently low status, its associations with OS and DFS also showed broadly consistent patterns across subgroups (**Supplementary Figure 4**). No significant interactions were observed, suggesting that the prognostic associations of mGRIm-related variables were relatively stable across clinically relevant subgroups.

### 3.5 Construction and validation of dynamic feature-based prognostic models

#### 3.5.1 Stable predictor selection using Boruta and resampling validation

**Figure 2:**
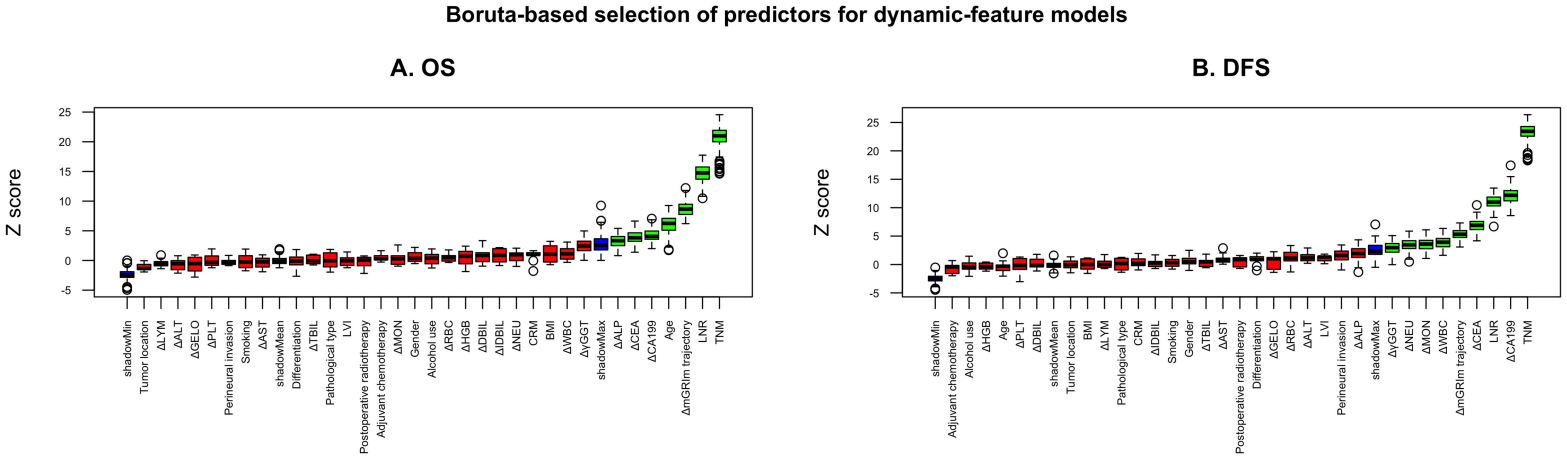
Boruta-based predictor selection for dynamic-feature models. Boruta feature selection was performed exclusively in the training set. **(A)** Selected predictors for OS prediction. **(B)** Selected predictors for DFS prediction. Green boxes indicate confirmed predictors, red boxes indicate rejected predictors, and blue boxes indicate shadow variables.

Boruta feature selection was performed exclusively within the training set using the dynamic feature space. For overall survival (OS) prediction, stable predictors included age, lymph node ratio (LNR), TNM stage, and perioperative dynamic biomarkers (ΔALP, ΔCEA, ΔCA199), together with the dynamic mGRIm trajectory. The Boruta-selected predictors are shown in **Figure 2** and summarized in **Supplementary Table 5**. For disease-free survival (DFS) prediction, stable predictors included lymph node ratio (LNR), TNM stage, and perioperative dynamic laboratory markers (ΔCEA, ΔCA199, ΔWBC, ΔMON, ΔNEU, and ΔγGGT), together with the dynamic mGRIm trajectory.

Importantly, predictor stability was further confirmed through 100 bootstrap resampling iterations. Features identified by the primary Boruta procedure demonstrated consistently high selection frequencies across resampling runs, supporting the robustness of the dynamic feature set. These findings indicate that both tumor burden-related variables and perioperative inflammatory dynamics contribute reproducibly to prognostic stratification. Detailed predictor selection frequencies are provided in **Supplementary Table 10**.

#### 3.5.2 Comparative performance of candidate machine-learning models

Using the Boruta-selected stable predictors, 13 machine-learning algorithms were trained under identical resampling conditions. All models were evaluated using a training set-restricted 5-fold cross-validation framework within the training set, with an independent test set reserved for final performance assessment.

For OS prediction, the neural network model achieved the highest composite score and demonstrated balanced performance across discrimination and classification metrics in the test set (AUC = 0.801, balanced accuracy = 0.730, F1 score = 0.542). Competing models, including multivariate adaptive regression splines, elastic net regression, and support vector machines, showed comparable but inferior overall performance, particularly in sensitivity and balanced accuracy.

For DFS prediction, the radial basis function support vector machine achieved the best composite score, with superior discrimination (AUC = 0.856) and balanced classification performance (balanced accuracy = 0.844). Other models showed less favorable sensitivity-specificity trade-offs, indicating differences in their ability to handle class imbalance.

#### 3.5.3 Integrated model selection and robustness interpretation

Final model selection was based on a prespecified composite metric integrating discrimination, balance-adjusted accuracy, and classification sensitivity. This composite framework was designed to mitigate the limitations of single-metric evaluation, particularly in the context of class-imbalanced clinical datasets.

**Figure 3:**
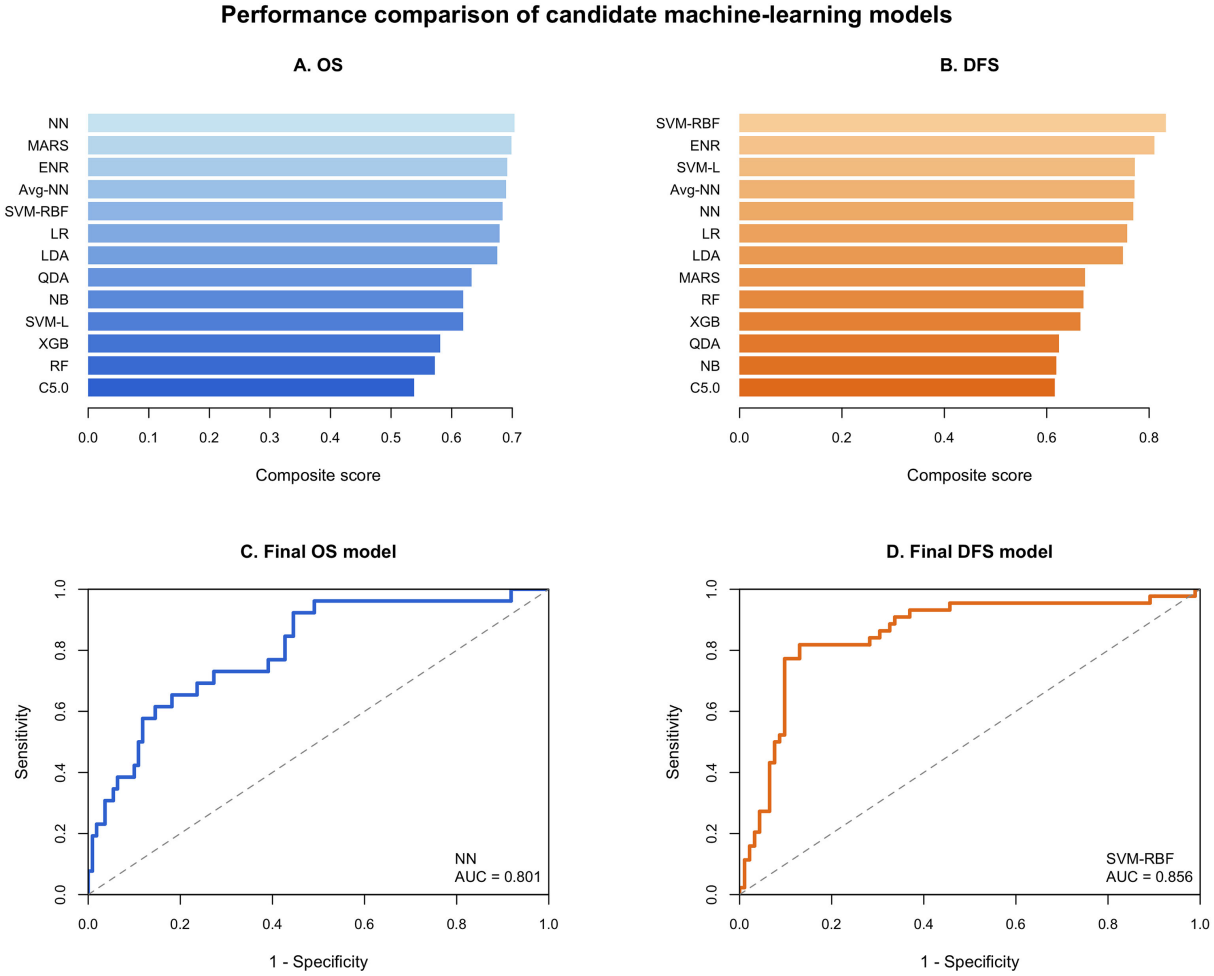
Performance comparison of candidate machine-learning models. Candidate models were trained using 5-fold cross-validation in the training set, with up-sampling applied within the resampling procedure to address class imbalance. The locked testing set was used only for final evaluation. **(A)** Composite score ranking for OS prediction. **(B)** Composite score ranking for DFS prediction. **(C)** Receiver operating characteristic curve of the final selected OS model. **(D)** Receiver operating characteristic curve of the final selected DFS model. NN, neural network; ENR, elastic net regression; Avg-NN, averaged neural network; SVM-RBF, support vector machine with radial basis function kernel; SVM-L, support vector machine with linear kernel; LR, logistic regression; LDA, linear discriminant analysis; QDA, quadratic discriminant analysis; NB, naive Bayes classifier; MARS, multivariate adaptive regression splines; RF, random forest; XGB, extreme gradient boosting; C5.0, C5.0 decision tree algorithm; ROC, receiver operating characteristic curve; AUC, area under the receiver operating characteristic curve; OS, overall survival; DFS, disease-free survival.

Accordingly, the neural network model was selected as the optimal OS prediction model, whereas the radial basis function support vector machine was selected for DFS prediction. Composite score rankings and receiver operating characteristic (ROC) curves are presented in **Figure 3**.

Overall, dynamic-feature-based machine-learning models showed promising predictive performance in the independent test set. Bootstrap resampling supported the stability of the Boruta-selected predictors. Nevertheless, because the study was based on a single-center retrospective cohort and included internal validation only, external validation is required before clinical translation.

## 4 Discussion

The systemic immune status of the host profoundly influences tumor biology by regulating angiogenesis, metastatic dissemination, immune surveillance, and therapeutic resistance (22–24). Within this framework, composite inflammation–nutrition–metabolism indices such as the GRIm score may capture multidimensional host–tumor interactions that are not fully reflected by single biomarkers, including SII, PNI, ALB, NLR, or conventional TNM staging (11, 12, 25–27).

Tumor-infiltrating and circulating immune cells, particularly lymphocytes and neutrophils, represent key effectors in the tumor microenvironment. Cytotoxic lymphocytes, including CD8+ T cells and natural killer cells, suppress tumor progression through direct cytolytic activity and are positively associated with immunotherapy responsiveness (28, 29). Conversely, reduced peripheral lymphocyte counts have been consistently associated with inferior survival outcomes in colorectal cancer (CRC) (30). In contrast, neutrophils contribute to tumor progression through multiple mechanisms, including immune evasion, angiogenesis, and metastatic facilitation (31). PD-L1–expressing tumor-associated neutrophils can further suppress T-cell function and promote immune escape (32). Clinically, elevated neutrophil-to-lymphocyte ratio (NLR) has been validated as an independent adverse prognostic marker in CRC, reflecting a systemic pro-inflammatory and pro-tumor state (33–35).

Beyond immune cell composition, systemic metabolic and nutritional status also plays a critical role in tumor progression. Lactate dehydrogenase (LDH), a key glycolytic enzyme, reflects tumor metabolic reprogramming and is associated with lactate accumulation, oxidative stress modulation, and oncogenic signaling activation (36, 37). Elevated LDH levels have been linked to poor prognosis in CRC, although its postoperative prognostic utility remains debated (38). Serum albumin serves as a surrogate marker of nutritional reserve and systemic physiological resilience, with hypoalbuminemia consistently associated with unfavorable oncologic outcomes (39). Importantly, inflammatory and nutritional dysregulation are biologically interconnected and may synergistically accelerate tumor progression (40). Accordingly, the GRIm score integrates inflammatory status (NLR), nutritional status (albumin), and metabolic activity (LDH), providing a composite reflection of host systemic homeostasis.

In the present study, we systematically evaluated the prognostic value of preoperative mGRIm, postoperative mGRIm, and their dynamic perioperative trajectories in CRC patients using retrospective cohort data. The results demonstrated that mGRIm-based stratification effectively discriminated survival risk across multiple time points, with the dynamic mGRIm trajectory providing the most robust prognostic stratification. Notably, the dynamic trajectory captures temporal evolution of inflammatory–metabolic status across the perioperative period, thereby offering additional prognostic resolution beyond single time-point measurements. This finding suggests that perioperative host systemic responses are not static but dynamically evolving processes that may better reflect tumor–host interaction intensity.

Multivariable Cox regression further confirmed the prognostic significance of mGRIm dynamics. A high preoperative mGRIm score was significantly associated with worse OS (HR = 1.96, p = 0.014) and DFS (HR = 1.86, p = 0.004), consistent with previous studies supporting the prognostic value of the GRIm score in solid tumors (25 -27). The postoperative mGRIm score demonstrated an even stronger association with OS (HR = 2.30, p < 0.001), potentially reflecting systemic inflammatory responses associated with surgical stress and early postoperative recovery (41). Importantly, the graded risk pattern observed across dynamic mGRIm trajectories further supports the concept that longitudinal biomarker evolution provides incremental prognostic information beyond static assessments.

To further evaluate the clinical utility of dynamic mGRIm-related features, we developed and compared multiple machine-learning models using a prespecified multidimensional evaluation framework. This framework integrated discrimination and imbalance-aware classification metrics to avoid reliance on a single evaluation criterion. Among 13 candidate algorithms, the neural network model achieved the highest composite score for OS prediction, while the radial basis function support vector machine performed best for DFS prediction. In the independent test set, both final models demonstrated satisfactory discrimination (OS: AUC = 0.801; DFS: AUC = 0.856), along with balanced classification performance. These results suggest that dynamic mGRIm trajectory, when combined with perioperative clinicopathological variables, provides a robust feature foundation for prognostic modeling. Differences in performance across algorithms underscore the value of comparing multiple candidate models rather than relying on a single modeling approach.

Several limitations should be acknowledged. (1) Single-center retrospective data: the data were obtained from a single medical center, Hubei Cancer Hospital affiliated with Huazhong University of Science and Technology, and may therefore be affected by selection bias and geographic bias, limiting the generalizability of the findings. In addition, the retrospective design could not fully control for confounding factors, such as treatment adherence and differences in postoperative care, which may affect the reliability of the conclusions. (2) Sample size and subgroup analysis: although 457 patients were included, the sample size in certain subgroups, such as stage IV patients and specific pathological subtypes, was small, resulting in limited statistical power to detect potential heterogeneity in associations. (3) Treatment heterogeneity: although information on adjuvant chemotherapy and radiotherapy was included, the analysis did not fully account for different chemotherapy regimens, dose adjustments, radiotherapy techniques, postoperative care, or treatment adherence, which may have influenced prognosis. (4) Limited time window for dynamic mGRIm trajectory: the current study focused only on perioperative dynamic mGRIm trajectory, from preoperative assessment to postoperative assessment before adjuvant therapy, and did not include long-term postoperative monitoring data, such as changes at 6 months or 1 year after surgery. Therefore, the prognostic implications of longer-term inflammatory-metabolic changes could not be comprehensively assessed. (5) Lack of external validation: although the final machine-learning models showed promising internal performance, they have not been validated in independent external cohorts, such as cohorts from other hospitals or ethnically diverse populations, and their generalizability requires further confirmation.

## 5 Conclusion

This study evaluated the prognostic value of preoperative mGRIm, postoperative mGRIm, and dynamic perioperative trajectories in colorectal cancer. mGRIm-based indicators were significantly associated with OS and DFS, with dynamic trajectories showing the strongest prognostic stratification. Patients with persistently high mGRIm status at both time points had the poorest outcomes (OS HR = 3.47, p < 0.001), indicating that longitudinal inflammatory–metabolic profiling provides superior prognostic resolution compared with single time-point assessment. Machine-learning models integrating dynamic mGRIm features and clinicopathological variables demonstrated satisfactory predictive performance, with neural networks and radial basis function SVM achieving the best results for OS and DFS, respectively. In conclusion, dynamic mGRIm trajectories may improve risk stratification in CRC. However, external validation is required before clinical application.

## Data Availability

The original contributions presented in the study are included in the article/**Supplementary Material**. Further inquiries can be directed to the corresponding author.
