# Supplementary material for: Construction of prognostic models for colorectal cancer based on the perioperative modified Gustave Roussy Immune Score and machine learning
Source: Front Immunol. 2026 Jul 30;17:1761650. doi: 10.3389/fimmu.2026.1761650 (PMC13468812; doi:10.3389/fimmu.2026.1761650)
Supplement: Supplementary file 1 [file SupplementaryFile1.docx]

Supplementary Material

# Construction of Prognostic Models for Colorectal Cancer Based on the Perioperative Modified Gustave Roussy Immune Score and Machine Learning

Jie Zhou^1,2^ , Kexin Zhao^4*^, Shaozhong Wei^2^, ShuaiJin Huang^3*^

^1^Department of Medical Oncology, Cancer Center, West China Hospital, Sichuan University, Chengdu, 610041, China.

^2^Department of Gastrointestinal Oncology Surgery, Hubei Cancer Hospital, Tongji Medical College, Huazhong University of Science and Technology, Wuhan 430079, Hubei, China

^3^Department of Emergency, Hunan Provincial Hospital of Integrated Traditional Chinese and Western Medicine, Changsha 410006, Hunan Province, China.

^4^Department of Hematology, The Second Xiangya Hospital, Central South University, Changsha, 410011, Hunan, China.

*** Correspondence:**
Kexin Zhao zkx162046@163.com

ShuaiJin Huang 14789868801@163.com

Table of Contents

Table S1. Preoperative characteristics stratified by survival and disease progression status.

Table S2. Postoperative characteristics stratified by survival and disease progression status.

Table S3. Dynamic characteristics stratified by survival and disease progression status.

Table S4. ROC-derived cut-off values for constructing preoperative and postoperative mGRIm scores.

Table S5. Boruta-selected predictors for dynamic-feature models.

Table S6. Performance comparison of candidate machine-learning models for OS prediction.

Table S7. Performance comparison of candidate machine-learning models for DFS prediction.

Table S8. Model selection criteria and final selected models.

Table S9. Sensitivity analyses for alternative definitions of perioperative mGRIm change.

Table S10. Stability of primary Boruta-selected predictors across repeated resampling.

Figure S1. Flowchart of patient selection.

Figure S2. Definition and grouping criteria for mGRIm score. Preoperative and postoperative mGRIm scores were calculated using LDH, ALB, and NLR according to ROC-derived cut-off values. Scores ≤1 were classified as low mGRIm status, whereas scores >1 were classified as high mGRIm status. Dynamic mGRIm trajectory was defined as group I, persistently low; group II, either preoperative or postoperative high; and group III, persistently high.

Figure S3. Subgroup analyses of preoperative and postoperative mGRIm status and prognosis. Forest plots show the associations of high versus low preoperative or postoperative mGRIm status with OS and DFS across predefined subgroups.

Figure S4. Subgroup analyses of dynamic mGRIm trajectory and prognosis. Forest plots show the associations of any high dynamic mGRIm trajectory versus persistently low trajectory with OS and DFS across predefined subgroups. OS, overall survival; DFS, disease-free survival; HR, hazard ratio.

Figure S5. Overview of the machine learning workflow for model development, validation, and final model selection for overall survival (OS) and disease-free survival (DFS) prediction.

Table S1. Preoperative characteristics stratified by survival and disease progression status.

|  | Survival Status | | p-value | Progress Status | | p-value |
| --- | --- | --- | --- | --- | --- | --- |
|  | Alive (N=369) | Dead (N=88) |  | No (N=309) | Yes (N=148) |  |
| WBC, 10^9^/L | 5.99 (±1.95) | 6.08 (±2.04) | 0.730 | 5.85 (±1.87) | 6.34 (±2.14) | 0.014 |
| RBC, 10^12^/L | 4.22 (±0.57) | 4.07 (±0.55) | 0.018 | 4.23 (±0.57) | 4.12 (±0.56) | 0.047 |
| PLT, 10^9^/L | 244.04 (±83.04) | 227.47 (±86.39) | 0.096 | 243.94 (±81.74) | 234.41 (±88.04) | 0.256 |
| HGB, g/L | 122.45 (±23.80) | 118.53 (±24.86) | 0.170 | 122.28 (±24.30) | 120.47 (±23.49) | 0.453 |
| NEU, 10^9^/L | 3.89 (±1.78) | 4.15 (±1.92) | 0.225 | 3.75 (±1.62) | 4.33 (±2.09) | 0.001 |
| MON, 10^9^/L | 0.44 (±0.17) | 0.47 (±0.21) | 0.196 | 0.43 (±0.17) | 0.48 (±0.19) | 0.005 |
| LYM, 10^9^/L | 1.53 (±0.56) | 1.33 (±0.54) | 0.002 | 1.54 (±0.56) | 1.40 (±0.56) | 0.016 |
| ALT, U/L | 21.99 (±18.11) | 22.94 (±15.13) | 0.650 | 22.45 (±19.18) | 21.59 (±13.62) | 0.626 |
| AST, U/L | 23.02 (±12.28) | 26.65 (±16.76) | 0.021 | 23.18 (±12.92) | 24.83 (±14.09) | 0.216 |
| ALP, U/L | 76.25 (±26.76) | 82.87 (±48.39) | 0.082 | 75.38 (±23.86) | 81.99 (±44.47) | 0.039 |
| γGGT, U/L | 29.57 (±34.15) | 45.76 (±85.85) | 0.005 | 28.06 (±29.42) | 42.35 (±73.86) | 0.003 |
| GELO, g/L | 27.40 (±4.53) | 27.80 (±4.69) | 0.459 | 27.25 (±4.49) | 27.94 (±4.68) | 0.126 |
| TBIL, μmol/L | 11.85 (±4.99) | 13.43 (±8.32) | 0.021 | 12.03 (±5.24) | 12.42 (±6.85) | 0.501 |
| DBIL, μmol/L | 2.56 (±1.46) | 2.77 (±1.79) | 0.233 | 2.59 (±1.50) | 2.61 (±1.59) | 0.893 |
| IDBIL, μmol/L | 9.27 (±4.03) | 9.69 (±3.75) | 0.380 | 9.40 (±4.23) | 9.26 (±3.41) | 0.720 |
| CEA, μg/L | 35.21 (±167.47) | 71.37 (±220.73) | 0.089 | 21.78 (±125.09) | 84.76 (±253.35) | <0.001 |
| CA199, U/mL | 221.85 (±1485.10) | 651.70 (±1982.41) | 0.023 | 205.74 (±1585.95) | 511.07 (±1613.38) | 0.056 |
| mGRIm score |  |  | <0.001 |  |  | <0.001 |
| ≤ 1 | 330 (89.4%) | 61 (69.3%) |  | 282 (91.3%) | 109 (73.6%) |  |
| > 1 | 39 (10.6%) | 27 (30.7%) |  | 27 ( 8.7%) | 39 (26.4%) |  |

Table S2. Postoperative characteristics stratified by survival and disease progression status.

|  | Survival Status | | | p-value | Progress Status | | | p-value |
| --- | --- | --- | --- | --- | --- | --- | --- | --- |
|  | Alive (N=369) | | Dead (N=88) |  | No (N=309) | | Yes (N=148) |  |
| WBC, 10^9^/L | 5.36 (±2.05) | 5.97 (±2.75) | | 0.020 | 5.30 (±1.91) | 5.83 (±2.72) | | 0.018 |
| RBC, 10^12^/L | 4.78 (±13.49) | 3.93 (±0.70) | | 0.554 | 4.94 (±14.74) | 3.95 (±0.62) | | 0.415 |
| PLT, 10^9^/L | 233.13 (±83.37) | 238.59 (±83.46) | | 0.581 | 231.65 (±82.57) | 239.46 (±84.92) | | 0.349 |
| HGB, g/L | 115.90 (±19.89) | 113.45 (±17.42) | | 0.289 | 116.32 (±20.55) | 113.59 (±16.83) | | 0.161 |
| NEU, 10^9^/L | 4.38 (±20.84) | 4.10 (±2.73) | | 0.901 | 4.54 (±22.75) | 3.88 (±2.67) | | 0.728 |
| MON, 10^9^/L | 0.41 (±0.18) | 0.56 (±0.75) | | 0.001 | 0.40 (±0.18) | 0.51 (±0.59) | | 0.003 |
| LYM, 10^9^/L | 1.48 (±0.55) | 1.41 (±0.54) | | 0.288 | 1.49 (±0.56) | 1.42 (±0.51) | | 0.208 |
| ALT, U/L | 25.24 (±28.14) | 22.01 (±15.29) | | 0.299 | 25.14 (±29.77) | 23.55 (±16.35) | | 0.543 |
| AST, U/L | 22.81 (±12.33) | 26.33 (±20.60) | | 0.038 | 22.36 (±11.93) | 25.85 (±18.20) | | 0.015 |
| ALP, U/L | 81.71 (±46.29) | 100.76 (±64.34) | | 0.001 | 78.24 (±30.29) | 100.28 (±75.73) | | <0.001 |
| γGGT, U/L | 36.20 (±81.24) | 59.49 (±97.95) | | 0.021 | 30.77 (±30.02) | 61.38 (±141.20) | | <0.001 |
| GELO, g/L | 29.40 (±10.22) | 30.55 (±5.20) | | 0.305 | 29.39 (±11.01) | 30.11 (±4.87) | | 0.448 |
| TBIL, μmol/L | 11.29 (±4.42) | 12.34 (±5.40) | | 0.055 | 11.35 (±4.52) | 11.80 (±4.88) | | 0.330 |
| DBIL, μmol/L | 2.58 (±1.51) | 2.94 (±2.04) | | 0.061 | 2.54 (±1.21) | 2.88 (±2.26) | | 0.037 |
| IDBIL, μmol/L | 8.68 (±3.46) | 9.49 (±3.93) | | 0.055 | 8.77 (±3.63) | 8.97 (±3.43) | | 0.561 |
| CEA, μg/L | 25.39 (±168.30) | 113.51 (±362.44) | | 0.001 | 12.52 (±122.69) | 104.66 (±339.25) | | <0.001 |
| CA199, U/mL | 57.29 (±397.48) | 1032.90 (±3785.35) | | <0.001 | 25.79 (±160.74) | 703.14 (±2994.91) | | <0.001 |
| mGRIm score |  |  | | <0.001 |  |  | | <0.001 |
| ≤ 1 | 272 (73.7%) | 32 (36.4%) | |  | 234 (75.7%) | 70 (47.3%) | |  |
| > 1 | 97 (26.3%) | 56 (63.6%) | |  | 75 (24.3%) | 78 (52.7%) | |  |

Table S3. Dynamic characteristics stratified by survival and disease progression status.

|  | Survival Status | | p-value | Progress Status | | p-value |
| --- | --- | --- | --- | --- | --- | --- |
|  | Alive (N=369) | Dead (N=88) |  | No (N=309) | Yes (N=148) |  |
| ΔWBC, 10^9^/L | 1.55 (±1.78) | 2.21 (±2.65) | 0.005 | 1.44 (±1.62) | 2.16 (±2.54) | <0.001 |
| ΔRBC, 10^12^/L | 1.14 (±13.43) | 0.50 (±0.44) | 0.656 | 1.27 (±14.67) | 0.48 (±0.42) | 0.509 |
| ΔPLT, 10^9^/L | 58.58 (±56.37) | 68.24 (±50.86) | 0.142 | 57.70 (±57.21) | 66.16 (±51.23) | 0.127 |
| ΔHGB, g/L | 16.21 (±16.75) | 16.88 (±12.95) | 0.728 | 16.41 (±17.25) | 16.20 (±13.37) | 0.895 |
| ΔNEU, 10^9^/L | 2.50 (±20.75) | 2.02 (±2.48) | 0.827 | 2.60 (±22.66) | 2.01 (±2.48) | 0.756 |
| ΔMON, 10^9^/L | 0.15 (±0.13) | 0.26 (±0.67) | 0.003 | 0.14 (±0.13) | 0.22 (±0.52) | 0.013 |
| ΔLYM, 10^9^/L | 0.39 (±0.36) | 0.45 (±0.42) | 0.168 | 0.39 (±0.36) | 0.41 (±0.41) | 0.709 |
| ΔALT, U/L | 13.12 (±27.04) | 11.73 (±14.38) | 0.642 | 13.24 (±28.83) | 12.03 (±14.53) | 0.630 |
| ΔAST, U/L | 8.38 (±12.21) | 10.66 (±14.31) | 0.129 | 8.32 (±12.48) | 9.87 (±12.98) | 0.219 |
| ΔALP, U/L | 18.05 (±32.06) | 27.29 (±35.99) | 0.018 | 15.75 (±18.42) | 28.34 (±50.61) | <0.001 |
| ΔγGGT, U/L | 16.82 (±69.03) | 25.84 (±36.17) | 0.236 | 13.18 (±24.96) | 29.78 (±106.07) | 0.009 |
| ΔGELO, g/L | 4.32 (±9.90) | 4.28 (±3.34) | 0.971 | 4.42 (±10.74) | 4.10 (±3.17) | 0.724 |
| ΔTBIL, μmol/L | 3.40 (±3.32) | 4.66 (±7.69) | 0.019 | 3.43 (±3.42) | 4.10 (±6.20) | 0.139 |
| ΔDBIL, μmol/L | 1.01 (±1.33) | 1.37 (±1.67) | 0.031 | 0.98 (±1.21) | 1.28 (±1.74) | 0.034 |
| ΔIDBIL, μmol/L | 2.66 (±2.53) | 2.86 (±2.48) | 0.503 | 2.68 (±2.66) | 2.73 (±2.19) | 0.860 |
| ΔCEA, μg/L | 20.91 (±76.24) | 69.40 (±249.22) | 0.002 | 12.75 (±39.83) | 66.79 (±217.11) | <0.001 |
| ΔCA199, U/mL | 202.89 (±1445.35) | 758.29 (±2625.24) | 0.007 | 200.41 (±1567.09) | 538.30 (±2057.81) | 0.053 |
| Dynamic mGRIm trajectory |  |  | <0.001 |  |  | <0.001 |
| I | 253 (68.6%) | 27 (30.7%) |  | 220 (71.2%) | 60 (40.5%) |  |
| II | 96 (26.0%) | 39 (44.3%) |  | 76 (24.6%) | 59 (39.9%) |  |
| III | 20 ( 5.4%) | 22 (25.0%) |  | 13 ( 4.2%) | 29 (19.6%) |  |

Table S4. ROC-derived cut-off values for constructing preoperative and postoperative mGRIm scores.

| **Variable** | **Time point** | **Cut-off** | **AUC** | **Sensitivity** | **Specificity** | **Youden index** | **Risk direction** |
| --- | --- | --- | --- | --- | --- | --- | --- |
| ALB | Preoperative | 39.150 | 0.618 | 0.420 | 0.786 | 0.206 | Lower value indicates higher risk |
| LDH | Preoperative | 221.000 | 0.592 | 0.284 | 0.905 | 0.189 | Higher value indicates higher risk |
| NLR | Preoperative | 3.649 | 0.591 | 0.375 | 0.780 | 0.155 | Higher value indicates higher risk |
| ALB | Postoperative | 41.250 | 0.644 | 0.636 | 0.631 | 0.268 | Lower value indicates higher risk |
| LDH | Postoperative | 171.950 | 0.665 | 0.591 | 0.718 | 0.309 | Higher value indicates higher risk |
| NLR | Postoperative | 2.407 | 0.606 | 0.545 | 0.642 | 0.188 | Higher value indicates higher risk |

Note: The optimal cut-off values were determined by receiver operating characteristic curve analysis using the Youden index, with overall survival status as the classification outcome. ALB values below the cut-off and LDH/NLR values above the cut-off were assigned 1 point. ALB, albumin; LDH, lactate dehydrogenase; NLR, neutrophil-to-lymphocyte ratio; AUC, area under the receiver operating characteristic curve.

**Table S5. Boruta-selected predictors for dynamic-feature models.**

| **Endpoint** | **Selected features** |
| --- | --- |
| OS | TNM |
| OS | LNR |
| OS | Age |
| OS | ΔALP |
| OS | ΔCA199 |
| OS | ΔCEA |
| OS | Dynamic mGRIm trajectory |
| DFS | TNM |
| DFS | LNR |
| DFS | ΔWBC |
| DFS | ΔNEU |
| DFS | ΔγGGT |
| DFS | ΔMON |
| DFS | ΔCA199 |
| DFS | ΔCEA |
| DFS | Dynamic mGRIm trajectory |

Note: Candidate predictors included clinicopathological variables and perioperative dynamic laboratory changes. Boruta feature selection was performed exclusively in the training set to reduce information leakage.

**Table S6. Performance comparison of candidate machine-learning models for OS prediction.**

| **Rank** | **Model** | **Composite score** | **AUC** | **Accuracy** | **Sensitivity** | **Specificity** | **Precision** | **F1 score** | **Kappa** | **Balanced accuracy** | **Best tune** |
| --- | --- | --- | --- | --- | --- | --- | --- | --- | --- | --- | --- |
| 1 | Neural network | 0.704 | 0.801 | 0.801 | 0.615 | 0.845 | 0.485 | 0.542 | 0.418 | 0.730 | size=3; decay=0.1 |
| 2 | Multivariate adaptive regression splines | 0.699 | 0.772 | 0.787 | 0.654 | 0.818 | 0.459 | 0.540 | 0.406 | 0.736 | nprune=4; degree=1 |
| 3 | Elastic net regression | 0.692 | 0.778 | 0.794 | 0.615 | 0.836 | 0.471 | 0.533 | 0.404 | 0.726 | alpha=0.55; lambda=0.080 |
| 4 | Averaged neural network | 0.690 | 0.797 | 0.735 | 0.654 | 0.755 | 0.386 | 0.486 | 0.323 | 0.704 | size=1; decay=0.01; bag=FALSE |
| 5 | Support vector machine with radial basis function kernel | 0.684 | 0.790 | 0.794 | 0.577 | 0.845 | 0.469 | 0.517 | 0.388 | 0.711 | sigma=0.257; C=0.25 |
| 6 | Logistic regression | 0.679 | 0.786 | 0.787 | 0.577 | 0.836 | 0.455 | 0.508 | 0.375 | 0.707 | parameter=none |
| 7 | Linear discriminant analysis | 0.675 | 0.790 | 0.772 | 0.577 | 0.818 | 0.429 | 0.492 | 0.349 | 0.698 | parameter=none |
| 8 | Quadratic discriminant analysis | 0.633 | 0.722 | 0.801 | 0.500 | 0.873 | 0.481 | 0.491 | 0.367 | 0.686 | parameter=none |
| 9 | Naive Bayes classifier | 0.619 | 0.762 | 0.794 | 0.423 | 0.882 | 0.458 | 0.440 | 0.314 | 0.652 | fL=0; usekernel=FALSE; adjust=1 |
| 10 | Support vector machine with linear kernel | 0.619 | 0.720 | 0.765 | 0.500 | 0.827 | 0.406 | 0.448 | 0.301 | 0.664 | C=1 |
| 11 | Extreme gradient boosting | 0.581 | 0.744 | 0.787 | 0.346 | 0.891 | 0.429 | 0.383 | 0.256 | 0.619 | nrounds=50; max_depth=4; eta=0.4; gamma=0; colsample_bytree=0.6; min_child_weight=1; subsample=0.5 |
| 12 | Random forest | 0.572 | 0.759 | 0.787 | 0.308 | 0.900 | 0.421 | 0.356 | 0.231 | 0.604 | mtry=2 |
| 13 | C5.0 decision tree algorithm | 0.538 | 0.760 | 0.779 | 0.231 | 0.909 | 0.375 | 0.286 | 0.164 | 0.570 | trials=30; model=1; winnow=TRUE |

Note: Candidate models were trained using 5-fold cross-validation in the training set. Up-sampling was performed within the resampling procedure to address class imbalance. The locked test set was used only for final model evaluation. Composite score was calculated as 0.40 × AUC + 0.25 × balanced accuracy + 0.20 × F1 score + 0.15 × sensitivity.

**Table S7. Performance comparison of candidate machine-learning models for DFS prediction.**

| **Rank** | **Model** | **Composite score** | **AUC** | **Accuracy** | **Sensitivity** | **Specificity** | **Precision** | **F1 score** | **Kappa** | **Balanced accuracy** | **Best tune** |
| --- | --- | --- | --- | --- | --- | --- | --- | --- | --- | --- | --- |
| 1 | Support vector machine with radial basis function kernel | 0.833 | 0.856 | 0.853 | 0.818 | 0.870 | 0.750 | 0.783 | 0.672 | 0.844 | sigma=0.242; C=0.25 |
| 2 | Elastic net regression | 0.810 | 0.845 | 0.824 | 0.795 | 0.837 | 0.700 | 0.745 | 0.611 | 0.816 | alpha=0.1; lambda=0.088 |
| 3 | Support vector machine with linear kernel | 0.772 | 0.823 | 0.779 | 0.750 | 0.793 | 0.635 | 0.688 | 0.519 | 0.772 | C=1 |
| 4 | Averaged neural network | 0.771 | 0.839 | 0.809 | 0.682 | 0.870 | 0.714 | 0.698 | 0.558 | 0.776 | size=1; decay=0.01; bag=FALSE |
| 5 | Neural network | 0.769 | 0.834 | 0.809 | 0.682 | 0.870 | 0.714 | 0.698 | 0.558 | 0.776 | size=1; decay=0.1 |
| 6 | Logistic regression | 0.757 | 0.825 | 0.787 | 0.682 | 0.837 | 0.667 | 0.674 | 0.516 | 0.759 | parameter=none |
| 7 | Linear discriminant analysis | 0.749 | 0.822 | 0.787 | 0.659 | 0.848 | 0.674 | 0.667 | 0.510 | 0.753 | parameter=none |
| 8 | Multivariate adaptive regression splines | 0.675 | 0.807 | 0.750 | 0.477 | 0.880 | 0.656 | 0.553 | 0.385 | 0.679 | nprune=8; degree=1 |
| 9 | Random forest | 0.672 | 0.772 | 0.779 | 0.477 | 0.924 | 0.750 | 0.583 | 0.443 | 0.701 | mtry=4 |
| 10 | Extreme gradient boosting | 0.666 | 0.760 | 0.757 | 0.500 | 0.880 | 0.667 | 0.571 | 0.407 | 0.690 | nrounds=50; max_depth=1; eta=0.3; gamma=0; colsample_bytree=0.6; min_child_weight=1; subsample=0.5 |
| 11 | Quadratic discriminant analysis | 0.624 | 0.783 | 0.743 | 0.364 | 0.924 | 0.696 | 0.478 | 0.328 | 0.644 | parameter=none |
| 12 | Naive Bayes classifier | 0.619 | 0.812 | 0.743 | 0.318 | 0.946 | 0.737 | 0.444 | 0.310 | 0.632 | fL=0; usekernel=FALSE; adjust=1 |
| 13 | C5.0 decision tree algorithm | 0.616 | 0.706 | 0.721 | 0.455 | 0.848 | 0.588 | 0.513 | 0.321 | 0.651 | trials=10; model=1; winnow=TRUE |

Note: Candidate models were trained using 5-fold cross-validation in the training set. Up-sampling was performed within the resampling procedure to address class imbalance. The locked test set was used only for final model evaluation. Composite score was calculated as 0.40 × AUC + 0.25 × balanced accuracy + 0.20 × F1 score + 0.15 × sensitivity.

**Table S8. Model selection criteria and final selected models.**

| **Endpoint** | **Final selected model** | **Primary selection criterion** | **Composite score definition** | **Class imbalance handling** | **Validation strategy** |
| --- | --- | --- | --- | --- | --- |
| OS | Neural network | Highest composite score among candidate models | 0.40 × AUC + 0.25 × balanced accuracy + 0.20 × F1 score + 0.15 × sensitivity | Up-sampling performed within the 5-fold cross-validation procedure in the training set | Stratified 7:3 train-test split; locked test set used only for final evaluation |
| DFS | Radial basis function support vector machine | Highest composite score among candidate models | 0.40 × AUC + 0.25 × balanced accuracy + 0.20 × F1 score + 0.15 × sensitivity | Up-sampling performed within the 5-fold cross-validation procedure in the training set | Stratified 7:3 train-test split; locked test set used only for final evaluation |

Note: The final models were selected according to the prespecified composite score integrating discrimination and imbalance-aware classification metrics. OS, overall survival; DFS, disease-free survival; AUC, area under the receiver operating characteristic curve.

**Table S9. Sensitivity analyses for alternative definitions of perioperative mGRIm change.**

| **Definition** | **Endpoint** | **Category/Unit** | **HR (95% CI)** | **P value** | **Model adjustment** |
| --- | --- | --- | --- | --- | --- |
| Three-level dynamic mGRIm trajectory | OS | Group II vs Group I | 2.052 (1.198–3.515) | 0.009 | Fully adjusted model |
| Three-level dynamic mGRIm trajectory | OS | Group III vs Group I | 3.692 (1.833–7.438) | <0.001 | Fully adjusted model |
| Binary dynamic mGRIm trajectory | OS | Any high vs both low | 2.322 (1.392–3.874) | 0.001 | Fully adjusted model |
| Ordinal dynamic mGRIm trajectory | OS | Per one-level increase in trajectory | 1.935 (1.372–2.73) | <0.001 | Fully adjusted model |
| Signed mGRIm change | OS | Per 1-point increase in signed change | 1.055 (0.838–1.329) | 0.648 | Fully adjusted model |
| Absolute mGRIm change | OS | Per 1-point increase in absolute change | 0.953 (0.71–1.28) | 0.750 | Fully adjusted model |
| Three-level dynamic mGRIm trajectory | DFS | Group II vs Group I | 1.812 (1.222–2.686) | 0.003 | Fully adjusted model |
| Three-level dynamic mGRIm trajectory | DFS | Group III vs Group I | 2.231 (1.313–3.79) | 0.003 | Fully adjusted model |
| Binary dynamic mGRIm trajectory | DFS | Any high vs both low | 1.903 (1.311–2.762) | <0.001 | Fully adjusted model |
| Ordinal dynamic mGRIm trajectory | DFS | Per one-level increase in trajectory | 1.542 (1.198–1.985) | <0.001 | Fully adjusted model |
| Signed mGRIm change | DFS | Per 1-point increase in signed change | 1.139 (0.95–1.365) | 0.160 | Fully adjusted model |
| Absolute mGRIm change | DFS | Per 1-point increase in absolute change | 1.07 (0.852–1.345) | 0.560 | Fully adjusted model |

**Note:** The fully adjusted model included age, sex, smoking, alcohol use, BMI, differentiation, tumor location, pathological type, LNR, CRM, LVI, perineural invasion, TNM stage, ΔCEA, ΔCA199, postoperative radiotherapy, and adjuvant chemotherapy. The three-level dynamic mGRIm trajectory was defined as group I, both preoperative and postoperative mGRIm low; group II, either preoperative or postoperative mGRIm high; and group III, both preoperative and postoperative mGRIm high. Signed mGRIm change was calculated as postoperative mGRIm minus preoperative mGRIm. Absolute mGRIm change was calculated as the absolute value of signed mGRIm change. OS, overall survival; DFS, disease-free survival; HR, hazard ratio; CI, confidence interval.

**Table S10. Stability of primary Boruta-selected predictors across repeated resampling.**

| **Endpoint** | **Predictor** | **Selection count** | **Valid runs** | **Selection frequency (%)** |
| --- | --- | --- | --- | --- |
| DFS | TNM stage | 100 | 100 | 100 |
| DFS | LNR | 100 | 100 | 100 |
| DFS | ΔWBC | 100 | 100 | 100 |
| DFS | ΔNEU | 100 | 100 | 100 |
| DFS | ΔγGGT | 100 | 100 | 100 |
| DFS | ΔMON | 100 | 100 | 100 |
| DFS | ΔCA199 | 100 | 100 | 100 |
| DFS | ΔCEA | 100 | 100 | 100 |
| DFS | Dynamic mGRIm trajectory | 100 | 100 | 100 |
| OS | TNM stage | 100 | 100 | 100 |
| OS | LNR | 100 | 100 | 100 |
| OS | Age | 100 | 100 | 100 |
| OS | ΔALP | 100 | 100 | 100 |
| OS | ΔCA199 | 100 | 100 | 100 |
| OS | ΔCEA | 100 | 100 | 100 |
| OS | Dynamic mGRIm trajectory | 100 | 100 | 100 |

**Note:** Boruta stability was assessed using 100 bootstrap resampling runs within the training set. This table reports only predictors confirmed in the primary Boruta analysis. Selection frequency was calculated as the percentage of valid bootstrap runs in which each predictor was confirmed by Boruta after TentativeRoughFix.

**Supplementary Figures**

Figure S1. Flowchart of patient selection.

Figure S2. Definition and grouping criteria for mGRIm score. Preoperative and postoperative mGRIm scores were calculated using LDH, ALB, and NLR according to ROC-derived cut-off values. Scores ≤1 were classified as low mGRIm status, whereas scores >1 were classified as high mGRIm status. Dynamic mGRIm trajectory was defined as group I, persistently low; group II, either preoperative or postoperative high; and group III, persistently high.

Figure S3. Subgroup analyses of preoperative and postoperative mGRIm status and prognosis. Forest plots show the associations of high versus low preoperative or postoperative mGRIm status with OS and DFS across predefined subgroups.

Figure S4. Subgroup analyses of dynamic mGRIm trajectory and prognosis. Forest plots show the associations of any high dynamic mGRIm trajectory versus persistently low trajectory with OS and DFS across predefined subgroups. OS, overall survival; DFS, disease-free survival; HR, hazard ratio.

Figure S5. Overview of the machine learning workflow for model development, validation, and final model selection for overall survival (OS) and disease-free survival (DFS) prediction.
